# Glycyl Radical Enzyme-Associated Microcompartments: Redox-Replete Bacterial Organelles

**DOI:** 10.1128/mBio.02327-18

**Published:** 2019-01-08

**Authors:** Bryan Ferlez, Markus Sutter, Cheryl A. Kerfeld

**Affiliations:** a MSU-DOE Plant Research Laboratory, Michigan State University, East Lansing, Michigan, USA; b Department of Biochemistry and Molecular Biology, Michigan State University, East Lansing, Michigan, USA; c Environmental Genomics and Systems Biology and Molecular Biophysics and Integrated Bioimaging Divisions, Lawrence Berkeley National Laboratory, Berkeley, California, USA; University of Texas Health Science Center at Houston

**Keywords:** activating enzymes, bacterial microcompartments, glycyl radical enzyme-associated microcompartments, glycyl radical enzymes, iron-sulfur proteins, electron transfer

## Abstract

An increasing number of microbes are being identified that organize catabolic pathways within self-assembling proteinaceous structures known as bacterial microcompartments (BMCs). Most BMCs are characterized by their singular substrate specificity and commonly employ B_12_-dependent radical mechanisms. In contrast, a less-well-known BMC type utilizes the B_12_-independent radical chemistry of glycyl radical enzymes (GREs). Unlike B_12_-dependent enzymes, GREs require an activating enzyme (AE) as well as an external source of electrons to generate an adenosyl radical and form their catalytic glycyl radical. Organisms encoding these glycyl radical enzyme-associated microcompartments (GRMs) confront the challenge of coordinating the activation and maintenance of their GREs with the assembly of a multienzyme core that is encapsulated in a protein shell. The GRMs appear to enlist redox proteins to either generate reductants internally or facilitate the transfer of electrons from the cytosol across the shell. Despite this relative complexity, GRMs are one of the most widespread types of BMC, with distinct subtypes to catabolize different substrates. Moreover, they are encoded by many prominent gut-associated and pathogenic bacteria. In this review, we will focus on the diversity, function, and physiological importance of GRMs, with particular attention given to their associated and enigmatic redox proteins.

## Abstract (precis)

An increasing number of microbes are being identified that organize catabolic pathways within self-assembling proteinaceous structures known as bacterial microcompartments (BMCs). Most BMCs are characterized by their singular substrate specificity and commonly employ B_12_-dependent radical mechanisms.

## INTRODUCTION

A microbe’s survival in new and/or competitive environments can be strongly influenced by its metabolic flexibility. To this end, bacteria frequently exchange genes encoding segments or entire metabolic pathways (1–3). These horizontal gene transfers (HGTs) can expand a host’s metabolic capacity to conditionally extract energy from new, potentially chemically resilient, substrates and increase their fitness. A particularly illustrative example of HGT-based expansion of metabolic flexibility is demonstrated by the widespread distribution of bacterial microcompartments (BMCs). BMCs function as bacterial organelles and are composed of multiple enzymes surrounded by a selectively permeable proteinaceous shell. BMC loci encode not only the structural components of the organelle, but also ancillary proteins, such as transmembrane transporters for associated metabolites, regulators, and even cytoskeletal elements, presumably used to position the organelle subcellularly. BMC loci are therefore genetic modules that encode a metabolic module, the organelle, and the ancillary proteins to integrate it into the host’s metabolism. This compact organization likely facilitates the HGT, which is apparent by examining their distribution across bacterial phyla (4). Indeed a BMC locus was one of the first proposed examples of the emerging concept of HGT (5).

**FIG 1:**
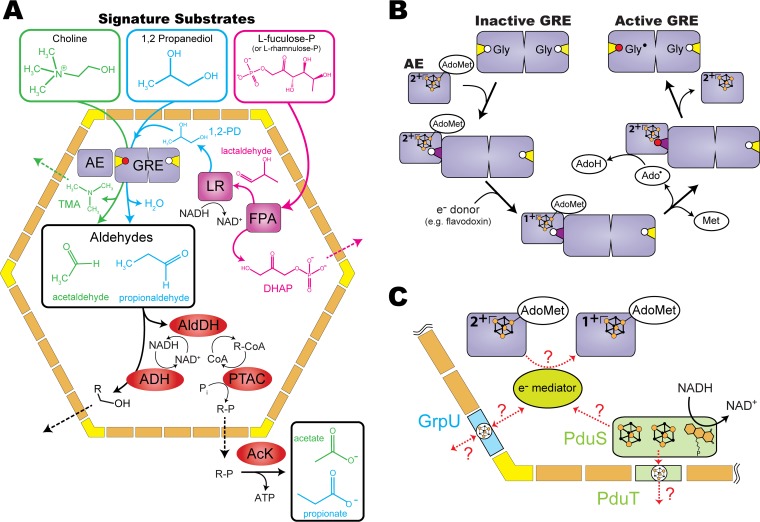
Overviews of GRM functional diversity and GRE activation. (A) Catabolic pathways for the three different substrates processed by distinct GREs encapsulated in GRMs: choline (green), 1,2-propanediol (1,2-PD) (cyan), and l-fuculose-P (or l-rhamnulose-P) (magenta and cyan). (B) GRE activation requires an activating enzyme (AE) and an external source of electrons. The active site glycine residue (white circle) located on the glycyl radical loop (closed conformation in yellow and open conformation in dark purple) is converted to a glycyl radical (Gly^·^ [red circle]) after hydrogen abstraction by the adenosyl radical (Ado^·^). Iron-sulfur (FeS) clusters are represented by orange (Fe) and black (sulfur) spheres. (C) Hypothetical redox reactions and electron transfer pathways involving PduS/PduT and/or GrpU. Abbreviations: ADH, alcohol dehydrogenase; AldDH, aldehyde dehydrogenase; PTAC, phosphotransacylase; AcK, acetate kinase; LR, lactaldehyde reductase; FPA, fuculose-P aldolase; DHAP, dihydroxyacetone phosphate. See the text for details.

Many catabolic BMCs, also known as metabolosomes (6), share a, paradigmatic, biochemistry, including a signature enzyme that degrades a specific substrate, thereby generating an aldehyde, and a series of aldehyde-processing enzymes (7) (Fig. 1A). This enzymatic core is surrounded by a protein shell, made up of three types of proteins that form cyclic oligomers (Fig. 1A): hexamers composed of BMC-H proteins (8), pseudohexameric trimers composed of BMC-T proteins (9), and pentamers composed of BMC-P proteins (10). Hexamers and trimers tile the facets of the shell (11) and have pores at their central cyclic axes of symmetry that mediate the transport of substrates and products into and out of the BMC. Pentamers cap the vertices of these polyhedral bodies (10, 11). As the interface with the rest of cellular metabolism, the selective permeability of the shell plays a critical role by limiting cross talk with other pathways, sequestering potentially toxic aldehyde intermediates, and/or improving pathway flux (12, 13).

One large group of catabolic BMCs encapsulate a signature enzyme that belongs to the ancient (14) and metabolically diverse glycyl radical enzyme (GRE) family (14, 15). These glycyl radical enzyme-associated microcompartments (GRMs) are further broken down into subclasses, depending on which distinct substrate is catabolized: choline, which is converted to acetaldehyde by elimination of trimethylamine (TMA) by a choline-TMA lyase GRE (16) (Fig. 1A, green); 1,2-propanediol (1,2-PD), which is dehydrated to propionaldehyde by a 1,2-PD dehydratase GRE (Fig. 1A, blue) (17); or l-fuculose-phosphate and l-rhamnulose-phosphate, which are metabolized to propionaldehyde and dihydroxyacetone phosphate (DHAP) by the concerted functions of an encapsulated fuculose-phosphate aldolase (FPA), lactaldehyde reductase (LR), and 1,2-PD dehydratase GRE (Fig. 1A, magenta and blue) (18, 19). These substrates are common breakdown products of glycans or lipids in the intestinal mucosa, and our growing appreciation of their significance in a healthy human gut microbiome underscores the impact GRM-mediated catabolism has on human health (3, 20). However, despite their contribution to the fitness of these gut microbes as well as the hosts’ health, the GRMs are relatively poorly understood.

GREs are typically homodimeric and lack any bound cofactors (21). Instead they use a stable glycyl radical (Gly^·^), along with a transient cysteine-based thiyl radical, located on the backbone of their glycyl radical domain, to catalyze their difficult chemical transformations (reviewed in reference 15). The Gly^⋅^ is produced posttranslationally by a GRE-specific activase, known as the activating enzyme (AE), which belongs to the radical *S*-adenosylmethionine (AdoMet) superfamily (22). AdoMet binds to a unique iron atom of the active site [4Fe4S]^2+^ cluster of the AE, which is reduced to the [4Fe4S]^1+^ state by an electron donor (e.g., flavodoxin). If the GRE substrate is also bound to the AE-AdoMet complex and its glycyl radical domain is in an open conformation, placing the active site glycine residue in close proximity to the AE iron-sulfur (FeS) cluster (23, 24), the bound AdoMet is next converted to the reactive adenosyl radical (Ado^⋅^) by direct electron transfer from the reduced [4Fe4S]^1+^ cluster. At this point, Ado^⋅^ can abstract a hydrogen atom from the active-site glycine residue of the GRE (Fig. 1B) (21). The resulting Gly^⋅^ is extremely sensitive to O_2_, which can inactivate the GRE by cleavage of its polypeptide (25). In the absence of O_2_, however, the Gly^⋅^ is remarkably stable, possibly due to further conformational changes of the glycyl radical loop following activation that shield the radical (23) (Fig. 1B); in the case of pyruvate-formate lyase (PFL) (a GRE which is not found in BMCs), the Gly^⋅^ half-life is >24 h under anoxic conditions (26). Finally, because the Gly^⋅^ is regenerated at the end of each catalytic cycle, each activated GRE can process multiple turnovers (15). Current mechanistic details of microcompartment-associated GREs have largely been studied outside the context of their native metabolic modules, and reconciliation with the temporal and spatial constraints of compartmentalization is an important challenge at the intersection of BMC and GRE biology.

Indeed, GRMs represent one of the largest, understudied, types of BMC. They catalyze a uniquely diverse array of reactions using glycyl radical chemistry; this versatility is likely to increase with the identification of new GREs. As our understanding of these complicated microbial metabolic modules advances, so too will our appreciation for their native, as well as potentially engineered, impact on the environment and human health. To this end, a few recent studies (3, 16, 18–20, 27) have raised awareness of the importance of GREs and the difficult chemical transformations they execute. However, the requirement for an AE as well as an electron donor to reduce its active site FeS cluster, prompts questions about how compartmentalization impacts the activation and function of GREs. In addition, our understanding of the putative redox proteins and electron transfer pathways associated with the GRMs remain relatively unexplored and represent an important target for investigation (Fig. 1C). Here we review the distribution, function, and internal organization of the GRM as a metabolic module as well as discuss its mechanistic requirements and the possible role of accessory FeS proteins in electron transfer reactions. Finally, we briefly discuss potential engineering applications that will benefit from a thorough understanding of GRM metabolism, particularly the associated electron transfer reactions.

## GRM DIVERSITY AND ABUNDANCE OF ACCESSORY FeS PROTEINS

**FIG 2:**
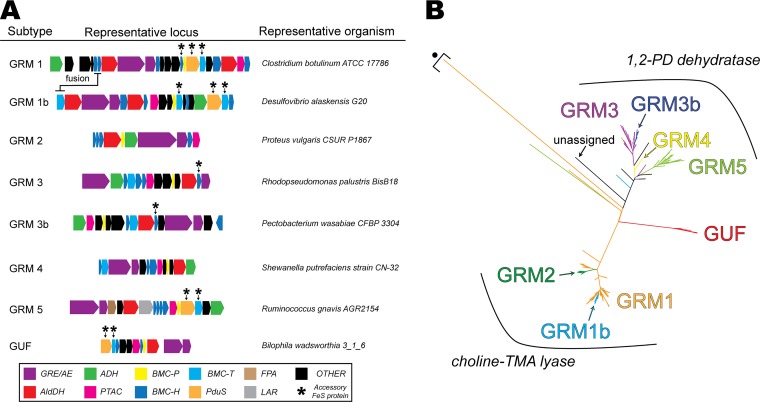
GRM locus variability, distribution of accessory FeS proteins, and GRE phylogeny. (A) Diagrams of loci for representative organisms encoding each GRM subtype. The accessory FeS proteins PduS, PduT, and GrpU are marked with black asterisks. (B) Phylogenetic tree of GRM GRE amino acid sequences showing they cluster predominantly by both subtype (GRM1, orange; GRM1b, light blue; GRM2, dark green; GRM3, purple; GRM3b, dark blue; GRM4, yellow; GRM5, light green; GUF, red; unassigned, black) and function (GRM1, -1b, and -2 genes encode choline-trimethylamine lyases; GRM3, -3b, -4, and -5 genes encode 1,2-PD dehydratases). Examples of GRM1 and GRM5 GREs that do not cluster with other sequences from their respective subtypes are marked by a black dot and square bracket.

GRM loci are distinguished from other BMC loci by the presence of genes encoding a GRE (pfam01228 and pfam02901) and a cognate AE (pfam04055). At the time this review was written, 536 GRM loci could be identified in the UniProt database (www.uniprot.org) and, based on their gene content (4), could be classified as belonging to one of six different subtypes: GRM1, GRM2, GRM3, GRM4, GRM5, or GUF (GRM of unknown function) (Fig. 2A). In addition, two instances of subtle differentiation based on the locus arrangement and gene sequences were observed within GRM1 and GRM3 subtypes, which we have named GRM1b and GRM3b. Finally, more than half of all GRM loci (348/536 [∼65%]) contain at least one accessory FeS protein—PduS/PduT and/or GrpU (see Potential Electron Transfer Mechanisms Inside and Across the Shell), hinting at their importance in GRM metabolism (Fig. 2A).

Consistent with previous bioinformatic analyses (3, 4, 19), a phylogenetic tree constructed from the amino acid sequences of GRM GREs shows that they cluster according to both their GRM subtype as well as biochemical function based on experimentally characterized representatives (Fig. 2B). GRM1 and GRM2 subtypes encode a choline-TMA lyase, GRM3, GRM4, a 1,2-PD dehydratase, and GRM5, a 1,2-PD dehydratase, along with an upstream fuculose-phosphate-aldolase (FPA [pfam00596]) and a lactaldehyde reductase (LR [pfam00107 and pfam08240]) for l-fuculose-P (or l-rhamnulose-P) degradation. Predicting the function of a newly identified GRE is difficult given that GREs possess a common structural architecture (28) and activation mechanism, despite catalyzing diverse chemical reactions (15, 29). For these reasons, the GUF loci, recently identified by Zarzycki et al. (19), await experimental characterization before they can be functionally classified.

## GRE SIGNATURE ENZYMES AND POTENTIAL ACCESSORY FeS PROTEINS

### Choline-trimethylamine lyase.

Microbial degradation of choline into TMA and acetaldehyde is an anaerobic metabolic activity that can be a source of both energy (ATP) and carbon (TMA and acetate). TMA also serves as an important substrate for neighboring archaeal methanogens in marine and ruminant gastrointestinal environments (30, 31). Moreover, because choline is an essential nutrient for humans and other vertebrates (32), its use by bacteria in the human gut has been linked to both human health and disease (33). The conversion of choline into TMA and acetaldehyde requires the energetically demanding cleavage of its C–N bond (15). Enzymes catalyzing similar elimination reactions involving C–N (ethanolamine ammonia lyase) or C–O (glycerol dehydratase) bonds typically require low-potential reactive radical species such as the Ado^⋅^ derived from adenosylcobalamin in B_12_-dependent enzymes (34). In contrast, the first GRE responsible for catalyzing the cleavage of the C–N bond of choline using a Gly^⋅^ was identified in 2012 (16). This GRE was recognized to be part of a larger conserved choline utilization (*cut*) gene cluster that also contains genes for BMC shell proteins as well as core aldehyde-processing enzymes common to catabolic BMCs (i.e., AldDH, ADH, and PTAC) (3, 4, 16, 19) (Fig. 1A). *cut* gene clusters were further subdivided into two groups, type I and type II (3), based on their gene content and the form of the signature GRE encoded by the *cutC* gene. This classification correlates with the nomenclature of GRM1 and GRM2 subtypes, respectively (4, 19).

Accessory FeS proteins are abundant in GRM1 loci: ∼85% encode a copy of the FeS BMC-H protein GrpU, and ∼25% encode the FeS proteins PduT and PduS (Fig. 2A). In contrast, all GRM1b loci encode homologs of PduT and PduS (Fig. 2A), but only one (out of 14 total) encodes a GrpU protein. A unifying feature of all GRM1 and GRM1b loci is the short N-terminal extension found on their GREs that is predicted to function as an encapsulation peptide (EP) to promote attachment to the luminal face of the BMC shell and/or facilitate the assembly of the microcompartment core (see Clues to Internal Organization of GRM GREs and AEs below) (19). In contrast to GRM1 and GRM1b, all GRM2 loci lack genes encoding PduS, PduT, or GrpU proteins. In addition, GRM2 GREs also lack a short putative N-terminal EP and instead have a much larger (∼350-amino-acid) N-terminal extension that resembles the N-terminal region of the GRM1 GREs (19). This extension is likely not functional as it lacks the glycyl radical loop containing the catalytic glycine and cysteine residues; however, it may play a role in encapsulation or organization (see Clues to Internal Organization of GRM GREs and AEs).

Tracking deuterated TMA (d_9_-TMA) production using high-performance liquid chromatography coupled to mass spectrometry (LC-MS) provides an unambiguous marker for choline-TMA lyase activity when d_9_-choline is provided as a substrate. Using this approach, heterologous expression of both CutC and CutD (the AE) from Desulfovibrio alaskensis G20 was necessary and sufficient to confer the ability to produce deuterated TMA (d_9_-TMA) from (trimethyl-d_9_)-choline in Escherichia coli (16). Moreover, this method confirmed choline-TMA lyase activity in a number of human gut isolates containing either GRM1 or GRM2 loci (3), consistent with their bioinformatically predicted function (3, 4, 19). These studies provided the first biochemical evidence linking choline degradation to bacteria containing GRM2 loci and have been further validated by knockout and complementation studies of the specific GRM2 GREs from Proteus mirabilis DSM4479 (35) and E. coli 536 (36).

### 1,2-Propanediol dehydratase.

1,2-PD is a common by-product of fucose and rhamnose degradation (37, 38) and can be an abundant source of energy and carbon for microbes in environmental niches such as the human gastrointestinal tract (39). 1,2-PD is the signature substrate of the well-studied propanediol utilization (PDU) BMC (40) and is dehydrated to propionaldehyde by the B_12_-derived Ado^·^ of the 1,2-PD dehydratase signature enzyme (34, 41). In contrast, GRM subtypes 3 (17), 4 (4, 19), and 5 (18, 27) catalyze a B_12_-independent dehydration of 1,2-PD using the protein-based glycyl/thiyl radicals of a GRE (34). To date, this is the only known example of functional redundancy among different types of catabolic BMCs.

As in the GRM1 loci, accessory FeS proteins are also abundant in GRMs containing 1,2-PD GREs. Approximately two-thirds (47/65) of GRM3 loci encode a PduT or PduS homolog, whereas only a few (3/65) encode a GrpU homolog (Fig. 2A). This distribution is reversed in GRM3b loci: less than one-third (14/65) encode a PduT or PduS homolog, but almost all (53/55) encode GrpU. The GRM4 locus lacks PduT, PduS, and GrpU (Fig. 2A), but it does encode a homolog of PduK, a poorly characterized BMC-H protein that contains a tetracysteine motif located on a C-terminal extension that may ligate an FeS cluster (42). As PduK homologs are rare in GRM loci (4/536), they will not be discussed further. All GRM5 loci encode PduT and PduS homologs (57/57), but none encode a GrpU homolog. Finally, all 1,2-PD dehydratase GREs from GRM3, GRM4, and GRM5 loci lack the N-terminal EP or extension observed in GRM1 or GRM2. Instead, they possess a unique ∼60-amino-acid insertion that is predicted to function as an EP (see Clues to Internal Organization of GRM GREs and AEs) (17, 19).

Currently, reports of functional studies of GRM3, GRM4, and GRM5 GREs are sparse; however, *in vitro* biochemical assays have confirmed the conversion of 1,2-PD to propionaldehyde by GREs from the GRM3 locus in Rhodopseudomonas palustris BisB18 (17) and the GRM5 locus in Roseburia inulinivorans (27). In addition to their GRE, GRM5 loci also encode two putative biochemically upstream enzymes to convert l-fuculose-P or l-rhamnulose-P into 1,2-PD: an FPA would convert l-fuculose-P (or l-rhamnulose-P) into lactaldehyde and DHAP, and an LR would reduce lactaldehyde to 1,2-PD at the expense of NADH (Fig. 1A) (18, 19). Consistent with this model, cultures of Clostridium phytofermentans grown in the presence of fucose or rhamnose produced both polyhedral BMC-like structures as well as fermentation products consistent with their predicted function (e.g., propanol and propionate) (18). The encapsulation of both fucose/rhamnose and 1,2-PD metabolic activities into a single BMC can be thought of as a partially condensed version of the larger subcellular metabolic activity found in Salmonella enterica: S. enterica first converts l-fuculose-P and l-rhamnulose-P to 1,2-PD and DHAP using the nonencapsulated fucose utilization pathway and then secretes 1,2-PD, which is ultimately taken back up and fermented by the B_12_-dependent PDU BMC (37, 39).

## CLUES TO INTERNAL ORGANIZATION OF GRM GREs AND AEs

Encapsulation peptides, short sequence extensions that are predicted to fold into alpha helices, but lack a conserved primary structure, are a distinguishing feature of a number of enzymes found in BMCs (43). They are typically fused to the N or C terminus (44) via a poorly conserved linker region of variable length (43). Encapsulation of proteins containing EPs, such as the signature enzymes of the PDU (45) and ethanolamine utilization (EUT) (46) BMCs, is thought to occur in part through interactions between the hydrophobic face of their EPs and the inside surface of the shell (47, 48). Moreover, EPs may also play a role in the biogenesis of the metabolosome core by facilitating the aggregation of encapsulated enzymes; removal of EPs often improves solubility of recombinant enzymes (49–54), whereas their addition can promote aggregation (55).

Interestingly, not all core enzymes contain EPs. For example, protein-protein interactions have been observed for both the EP-containing AldDH and EP-lacking ADH (56) as well as the B_12_-recycling enzymes PduO (adenosyltransferase) and PduS (corrin reductase) of PDU BMCs that likely lead to encapsulation via “piggy-backing” (57). In the latter case, although no EPs have been identified for either PduO or PduS, PduS interacts directly with the shell protein PduT via its N terminus (see Potential Electron Transfer Mechanisms Inside and Across the Shell) (58). These data suggest that the PduO/PduS complex is encapsulated via an EP-independent manner through protein-protein interactions with the shell. Together these results support a model of BMC biogenesis and organization that involves a combination of protein-protein interactions between different encapsulated enzymes as well as between enzymes and the shell using both EP-dependent and -independent mechanisms. This model is reminiscent of the experimentally validated core-first assembly of the anabolic β-carboxysome BMC (59).

A picture of the internal organization of GRM BMCs can be pieced together based on bioinformatic and experimental observations. The GRE signature enzymes of GRM1 (19), GRM3, GRM4, and GRM5 are all predicted to contain EPs (17, 19, 60). A notable exception is the GRM2 GREs, which appear to lack a canonical EP. However, the large ∼350-amino-acid N-terminal extension, which shares sequence similarity with the N terminus of the choline-TMA lyase enzymes from GRM1 loci, may function as either a nontraditional EP by interacting directly with the luminal surface of the shell and/or as an assembly factor by tethering multiple GREs together (19). The idea that the N-terminal extension might serve a role in GRM assembly by coalescing GREs is interesting given that many GREs, including the recently crystallized GRM1 choline-TMA lyase (49, 50), form homodimers via interactions involving N-terminal residues. The noncatalytic N-terminal extension of the GRM2 GRE therefore potentially adds an additional dimerization interface capable of interacting with either another N-terminal extension and/or a catalytically active domain of a second GRE. Expansion of such interactions beyond a pair of GREs could lead to either a serial linkage or aggregation of enzymes that could be important for packaging within the GRM. Unfortunately, crystallization of the GRM2 GRE from Klebsiella pneumoniae was only successful following protease treatment that removed this large N-terminal extension (61), so the structure of this extension remains elusive. Furthermore, a number of crystal structures of dimeric GREs also suggest the possible formation of tetrameric assemblies (dimers of dimers) that, although not always present in solution (50), could be relevant when concentrated within the GRM lumen (49). This raises interesting questions: How are GREs packaged within a GRM? In GRM2 compartments, does dimerization occur between the N-terminal extension and the main catalytic domain of the GRE? How do compartmentalization and oligomerization affect activation, stability, and/or turnover of GREs? Perhaps related to these questions is the longstanding, and as yet unexplained, observation that dimeric GREs only contain one Gly^⋅^ per GRE homodimer as opposed to two, suggesting only a single monomer is active (15). How this potential half-site reactivity may relate to organization within the GRM lumen, particularly in GRM2 BMCs, is an open question. No biophysical characterization of GRM GREs within the context of a microcompartment has been reported, and evidence of higher-order or alternate oligomeric states as well as their influence on activity remains to be determined.

Compartmentalization of GREs is further complicated by the strict requirement of an FeS cluster-containing AE for the posttranslational activation of the catalytic Gly^⋅^. Unlike the self-sufficient B_12_-dependent formation of the Ado^⋅^ by homolytic cleavage of adenosylcobalamin in the PDU and EUT compartments, GRE-AEs require a stoichiometric source of electrons from an external donor in order to generate Ado^⋅^. This necessity for reducing equivalents, as well as the lack of any identifiable EPs on any GRM AE, also raises questions about the spatial organization and temporal activation of GRM GREs: Does GRE activation take place prior to or after complete assembly of the GRM? If activation occurs before assembly, how is encapsulation of unactivated GREs minimized? If activation occurs after assembly, how are AEs encapsulated and what are the source and sequence for the reduction of their active site FeS clusters?

At this point, structural (23) and biochemical information on GRE-AE complexes is limited primarily to studies involving the AE of the well-characterized PFL-GRE (which has not been identified in BMCs) due to challenges in stability, oxygen sensitivity, and FeS cluster homogeneity of heterologous preparations (15, 21). However, from these studies we predict that binding of an AE to its cognate GRM GRE likely involves an analogous conformational change in the glycine radical loop region that places the active site glycine residue in close proximity with the AE active site FeS cluster and source of Ado^·^. In addition, binding of AdoMet to the AE is likely an independent event (62), but its reduction to the Ado^·^ requires an AE-GRE complex (63). In E. coli, the electron donor to PFL-AE is flavodoxin (Fld) (64, 65), and its binding site is distinct from the PFL/PFL-AE interface (23, 66). In combination with the experimentally measured (*K_d_*) dissociation constant values for Fld:PFL-AE, PFL:PFL-AE, and AdoMet:PFL-AE, these data suggest that *in vivo*, 90% of PFL is in complex with PFL-AE bound to AdoMet; of these PFL/PFL-AE complexes, only ∼11% also possess a bound Fld (62). Therefore, it is feasible that in GRMs, AEs are encapsulated by “piggy-backing” along with their EP-possessing GREs. This is supported by recent *in vitro* pulldown experiments that confirmed an interaction between the recombinant GRE and AE from a GRM3 locus (17). Likewise, extrapolation from the PFL/PFL-AE studies suggests that a substoichiometric number of Fld proteins might also become encapsulated during biogenesis of GRM BMCs. Even a small number of Flds, once confined to the GRM lumen, would likely be able to activate a larger number of GRE-AE complexes as long as (i) the binding sites on other AEs were accessible and (ii) either internal (within the GRM lumen) or external (from the cytosol and across the shell) electron transfer pathways existed to support their reduction.

One important difference between the AEs for PFL and the majority of GRM GREs (as well as many other non-GRM AEs) is the insertion of a small ∼70-amino-acid ferredoxin-like domain (pfam00037) in the latter (only GRM5 AEs lack this insertion). This domain is rich in cysteine residues and may be responsible for ligating one or more auxiliary FeS clusters (reviewed in reference 67). Direct evidence for the presence of additional clusters in GRM AEs comes from the iron content of purified, chemically reconstituted, recombinant AEs from both a GRM1 (∼8.4 mol of iron per protein) (50) and a GRM3 locus (13.1 mol of iron per protein) (17). Currently, data on these auxiliary FeS clusters are limited, and their numbers, identities, and functions have yet to be unequivocally established.

## POTENTIAL ELECTRON TRANSFER MECHANISMS INSIDE AND ACROSS THE SHELL

If GRE activation takes place after complete assembly of the GRM shell, reduction of the active site FeS cluster of an encapsulated AE would be required. Although no direct evidence for electron transfer reactions involving encapsulated AEs exists, almost two-thirds of all GRM loci found in UniProt currently encode at least one accessory protein predicted to bind an FeS cluster. One example is the soluble flavoenzyme PduS, which in the case of the homologous protein from PDU BMCs, binds one molecule of FMN, two FeS clusters, can oxidize NADH (58), and interacts with the FeS BMC-T protein PduT (42, 68). Another accessory FeS protein predicted to be a constituent of the GRM shell is the BMC-H protein GrpU (69), which, like PduT, binds an FeS cluster in its central pore. The function of the recently identified PduS, PduT, and GrpU homologs in GRM BMCs remains an open question, but their prevalence in GRM loci and, in the case of PduS/PduT, homology to PDU proteins suggest they may play an important role in redox and/or electron transfer reactions (Fig. 1C).

### PduS.

PduS is an ∼49-kDa protein composed of four pfam domains (http://pfam.xfam.org): the 51-kDa domain of complex 1 from the respiratory electron transfer chain (pfam01512), a soluble-ligand binding β-grasp domain (SLBB [pfam10531]), a [4Fe4S] dicluster domain (Fer4_17 [pfam13534]), and the N-terminal sandwich barrel hybrid motif (SBHM) from a peripheral subunit of the Rhodobacter nitrogen fixation (Rnf) energy-transducing membrane complex (RnfC_N [pfam13375]) (57, 70). Within the PDU BMC, PduS is believed to localize to the luminal face of the shell as part of a complex with the FeS shell protein PduT (58) and the adenosyltransferase PduO (57). These proteins participate in the regeneration of the adenosylcobalamin cofactor used by the B_12_-dependent signature enzyme 1,2-PD dehydratase (58). The precise role of PduT in this complex is unknown, but it may serve as a conduit for the export of excess electrons from the PDU lumen (see below) (42, 68). Consistent with its predicted domain assignments, characterization of heterologous preparations of the PduS homolog from the PDU locus in Citrobacter freundii using LC-MS identified a noncovalently bound flavin mononucleotide (FMN) cofactor. In addition, optical and electron paramagnetic resonance spectroscopies indicate the presence of two FeS clusters (arbitrarily identified here as FeS_1_ and FeS_2_) as well as the NADH-dependent reduction of both FMN and one of these FeS clusters (58). Although the identity of the reduced cluster could not be determined, observation of a paramagnetic singly reduced flavosemiquinone species following incubation with NADH suggests FMN, like other redox active flavins (71), can initially accept two electrons from NADH and subsequently pass one on to an acceptor—in this case, an FeS cluster (58). PduS can also mediate the reduction of cob(III)alamin to cob(II)alamin independent of PduO, suggesting that it can coordinate cobalamin, perhaps through its SLBB domain (pfam10531) (70), and/or catalyze its reduction. Alternatively, because free flavins can mediate the reduction of unbound cob(III)alamin (72), as well as PduO-bound cob(II)alamin (73), the reduced FMN cofactor may dissociate from PduS before reducing cobalamin. In either case, the role of the FeS_1_ and FeS_2_ clusters bound by the [4Fe4S] dicluster domain (pfam13534) is unclear, although their presence has been observed to raise the midpoint potential of the FMN cofactor from –262 ± 5 mV (versus standard hydrogen electrode [SHE]) when measured in the absence of the FeS clusters to –150 ± 5 mV (versus SHE) when measured in their presence (58).

Despite its prominent role in the B_12_-dependent chemistry of PDU BMCs, *pduS* homologs are also a common component of GRM loci and are almost always encoded adjacent to a *pduT* gene (Fig. 2A). These PduS homologs also possess a glycine-rich motif and Rossman-like fold common to pfam1512 domains (70), suggesting they also bind NADH and FMN cofactors. Moreover, a sequence alignment of the [4Fe4S] dicluster domains (pfam13534) of PduS homologs from both GRM and PDU loci indicates the presence of two highly conserved tetracysteine motifs consistent with the ligation two FeS clusters. In light of the conservation of both the *pduS*/*pduT* gene arrangement as well as the NADH, FMN, and FeS binding sites, the question becomes, what is the function of a corrin reductase in the B_12_-independent metabolism of GRM BMCs? One hypothesis is that PduS acts as an NADH:flavodoxin oxidoreductase that extracts electrons from NADH for the reduction, via an encapsulated electron mediator such as flavodoxin, of the AE to support GRE activation (Fig. 1C). However, this hypothesis remains to be experimentally validated and will require (i) confirmation that flavodoxin is encapsulated within the GRM lumen and (ii) demonstration that PduS is capable of carrying out NADH-dependent flavodoxin reduction.

### PduT.

PduT is a BMC-T protein that oligomerizes into a pseudohexameric trimer with a conserved cysteine residue oriented toward the central pore located at its 3-fold axis of symmetry (42, 74). In PduT homologs from PDU loci, the conserved cysteine residues from each of the three protomers of the trimer act as a ligand to one of three iron atoms of a [4Fe4S] cluster (68); the position and ligand to the 4th unique iron site are unknown, and it could be oriented either toward the lumen of the BMC or the cytosol. PduT homologs found in GRM loci also contain this conserved cysteine residue (C-P/S/A-G-K/R/S-Y/F) and therefore also likely coordinate an FeS cluster in their pore. This conserved cysteine, the tandem gene arrangement with *pduS*, and the experimental evidence of *in vitro* interaction between PduT and PduS homologs suggest PduS and PduT could form an electron transfer complex connecting the lumen of GRM BMCs with the cytosol. In the absence of structural data and redox potentials for the FeS clusters of PduS, it is not clear how, if at all, electrons are transferred between the redox cofactors of PduS and PduT (Fig. 1C). However, the midpoint potential of the [4Fe4S] cluster of PduT from a PDU BMC has been measured to be +99 mV (versus SHE) (68) and is therefore suitably poised to serve as an electron acceptor for excess electrons derived from the oxidization of NADH (midpoint redox potential [*E_m_*] at pH 7 of –320 mV versus SHE) (75). The wide distribution and potentially promiscuous redox behavior of PduS/PduT complexes in PDU and GRM BMCs are intriguing and warrant further structural, biochemical, and genetic analyses.

### GrpU.

Even less is known about the BMC-H shell protein GrpU. GrpU is distinguished by the conserved G-X-C-P-Q-N/H residues (where X is a variable position) of its pore motif and therefore presumably contains six cysteines at the central pore of its hexamer. Although a recent crystallographic analysis of two GrpU homologs was unable to resolve the structure of this pore, a broad absorption feature at ∼420 nm was observed for both recombinant proteins initially following purification, suggestive of an FeS cluster (69). Mutation of the conserved cysteine residue in the pore led to a loss of the absorption feature at ∼420 nm. These results are consistent with the hypothesis that GrpU, like PduT, binds an FeS cluster in its pore. An important difference between PduT and GrpU, however, is the additional three cysteine residues, or potential Fe ligands, in the pore of the latter. How these structural differences influence the geometry and/or chemical properties of the FeS cluster, however, remains to be determined.

Unlike *pduS* and *pduT*, *grpU* is almost exclusively found in GRM loci, and the adjacent genes vary depending on the GRM locus type. This variability in gene arrangement surrounding *grpU* may reflect a different local structural organization within the GRM BMC. For example, GrpU may not act as a fixed subunit of a larger electron transfer complex, as is predicted for the tandemly encoded PduS and PduT. Instead it may provide an FeS cluster that is accessible for soluble electron mediators (e.g., flavodoxin) to transiently dock and either donate or accept electrons from the outside or inside face of the shell (Fig. 1C). The ability to interact with soluble electron transfer proteins as well as the redox properties, occupancy, and stoichiometry of the GrpU FeS cluster will be critical in determining the function of GrpU in GRM metabolism and to efforts to build electron transfer interfaces between BMCs and the cytosol.

### GRMs lacking the accessory FeS proteins PduS, PduT, and GrpU.

Although widespread, not all GRM loci contain accessory FeS proteins: approximately one-third of the GRM loci currently detectable in Uniprot lack PduS, PduT, and GrpU and represent primarily the GRM2 subtype. If accessory FeS proteins support AE activation, their absence from both the core (PduS) and shell (PduT and GrpU) proteins of GRM2 BMCs would suggest activation of their GREs precedes encapsulation in order to avoid the wasteful assembly of a nonfunctional GRM. If GRE activation can take place prior to encapsulation, why enlist accessory FeS proteins at all? Do these accessory FeS proteins facilitate the reduction of encapsulated AEs, or do they have an alternate function? At present, two observations stand out as potentially important for reconciling both the wide distribution of accessory FeS proteins and their apparent expendability in GRM2 BMCs. First, unlike most other GRM subtypes, GRM2 loci are found exclusively in facultative anaerobes. Therefore, the lack of accessory FeS proteins may be a functional adaptation to episodic O_2_ exposure; the FeS clusters of PduS (58), PduT (74), and GrpU (69) all appear to be sensitive to damage by O_2_. It is tempting to speculate that some population of all GRM GREs are initially activated via cytosolic electron transfer pathways (e.g., Fld and Fld:NADH oxidoreductase) before encapsulation, and only in a case such as the GRM2 are the rewards for maximizing this fraction of activated GREs postassembly, by using accessory FeS cluster proteins, for example, offset by the benefit of a more O_2_-tolerant microcompartment. Alternatively, there could be other unidentified factors contributing to the activation of GREs before and/or after encapsulation. The second important distinction between GRM2 and the other GRM subtypes is the unique N-terminal extension of its GRE. Given the extreme O_2_ sensitivity of activated GREs, their propensity to dimerize (and sometimes tetramerize), and the observation that GREs appear to harbor only one Gly^·^ per homodimer, it is possible that this unique N-terminal extension affects oxygen stability, activation, and/or organization of the GRE in ways that improve the encapsulation of functional enzymes during GRM biogenesis.

## BIOENGINEERING POTENTIAL OF GRMs

As we learn more about the internal organization of GRM GREs and the function of the accessory FeS proteins, we will gain important insight into the role of redox and radical reactions in the context of the GRM-confined chemistry. This provides the foundation for their ability to be redesigned as well as their potential for biotechnological implementation. For example, by leveraging our growing understanding of the encapsulation and activation of GREs in native systems, we can begin to design new GRMs that support the challenging and biotechnologically relevant chemical transformations catalyzed by other diverse members of the GRE family, such as the recently discovered toluene-producing GRE PhdB (29). By choosing to encapsulate new GRE-associated pathways, we may realize increased stability and functional enzyme titers by way of organizing enzymes within the lumen and shielding their catalytic radicals from otherwise irreversible inactivation by O_2_. In addition, compartmentalization may also allow for control over pathway flux by encapsulating additional upstream enzymes in a manner analogous to GRM5 BMCs that couple segments of both fucose/rhamnose and 1,2-PD metabolisms.

In parallel, an understanding of electron transfer across GRM shells could enable the repurposing or design of new electron transfer pathways to support non-GRE-based catalysis. Regardless of whether or not the shell proteins PduT and GrpU serve a role in electron transfer reactions within GRMs, the physical location and potential solvent accessibility of their FeS clusters from both cytosolic and luminal sides make them promising candidates for development of electron transfer relays into and out of a BMC. Toward this end, a BMC-T protein was recently engineered to bind an [4Fe4S] cluster in its pore by mutation of a pore-located serine residue to a cysteine. This engineered FeS cluster could reversibly cycle between oxidized and reduced states and has a midpoint potential of –370 mV (versus SHE), functionally distinguishing it from its PduT-inspired template (*E_m_* = +99 mV) and demonstrating the energetic flexibility of these FeS binding sites (76). Collectively, the properties of the GRMs suggest that insights obtained from working out the details of both their core metabolism and shell properties will enable design and assembly of synthetic BMCs that encapsulate, and potentially enhance, challenging redox chemistries that may even require posttranslational activation and/or protection from O_2_. Furthermore, control over the thermodynamics, organization, and electrical connections of these redox modules could lead to their “hard-wiring,” for example, within phototrophic metabolisms such that the energetic cost of their catalysis is met by unused or redirected photosynthetic output.
